# Mechanistic Study on the Nuclear Modifier Gene *MSS1* Mutation Suppressing Neomycin Sensitivity of the Mitochondrial 15S rRNA C1477G Mutation in *Saccharomyces cerevisiae*

**DOI:** 10.1371/journal.pone.0090336

**Published:** 2014-03-03

**Authors:** Qiyin Zhou, Wei Wang, Xiangyu He, Xiaoyu Zhu, Yaoyao Shen, Zhe Yu, Xuexiang Wang, Xuchen Qi, Xuan Zhang, Mingjie Fan, Yu Dai, Shuxu Yang, Qingfeng Yan

**Affiliations:** 1 College of Life Sciences, Zhejiang University, Hangzhou, China; 2 Sir Run Run Shaw Hospital, Zhejiang University School of Medicine, Hangzhou, China; 3 The Second Affiliated Hospital, Zhejiang University School of Medicine, Hangzhou, China; CNR, Italy

## Abstract

The phenotypic manifestation of mitochondrial DNA (mtDNA) mutations can be modulated by nuclear genes and environmental factors. However, neither the interaction among these factors nor their underlying mechanisms are well understood. The yeast *Saccharomyces cerevisiae* mtDNA 15S rRNA C1477G mutation (P^R^) corresponds to the human 12S rRNA A1555G mutation. Here we report that a nuclear modifier gene *mss1* mutation suppresses the neomycin-sensitivity phenotype of a yeast C1477G mutant in fermentable YPD medium. Functional assays show that the mitochondrial function of the yeast C1477G mutant was impaired severely in YPD medium with neomycin. Moreover, the *mss1* mutation led to a significant increase in the steady-state level of *HAP5* (heme activated protein), which greatly up-regulated the expression of glycolytic transcription factors *RAP1*, *GCR1*, and *GCR2* and thus stimulated glycolysis. Furthermore, the high expression of the key glycolytic enzyme genes *HXK2*, *PFK1* and *PYK1* indicated that enhanced glycolysis not only compensated for the ATP reduction from oxidative phosphorylation (OXPHOS) in mitochondria, but also ensured the growth of the *mss1*(P^R^) mutant in YPD medium with neomycin. This study advances our understanding of the phenotypic manifestation of mtDNA mutations.

## Introduction

The phenotypic expression of mitochondrial DNA (mtDNA) mutations can be modulated by nuclear genes and environmental factors [1]–[5]. In the yeast *Saccharomyces cerevisiae* the mtDNA 15S rRNA C1477G mutation corresponds to the human mtDNA 12S rRNA A1555G mutation [6]–[9]. In this ideal model of the C1477G mutant, we and others have shown that the presence of additional mutations in the nuclear modifier genes *MSS1*, or *MTO1*, or *MTO2* causes the double mutant to exhibit a respiratory-deficient phenotype [8], [10]–[14]. In addition, it has been shown that the yeast C1477G mutant is resistant to the aminoglycosides neomycin and paromomycin in a non-fermentable YPG medium. This mutation has therefore also been called P^R^ or P^R^_454_ (paromomycin resistance) mutation [15], [16]. Together, these studies identify the essential roles of both nuclear modifier genes and environmental factors in mediating the phenotypic manifestation of the yeast mtDNA C1477G mutation.

To date, the interaction among mtDNAs, nuclear genes and environmental factors remains poorly understood. Recently we demonstrated that the yeast C1477G mutant was sensitive to aminoglycoside neomycin in a fermentable YPD medium, and that the nuclear modifier gene *mto2* mutation suppressed the C1477G mutant's phenotypic expression [17]. In *Saccharomyces cerevisiae*, the products of *MTO2* and *MSS1* are involved in the biosynthesis of the hypermodified nucleoside 5-methyl-aminomethy-2-thio-uridine (mnm^5^S^2^U34) in the wobble position of mitochondrial tRNAs [18], [19]. MTO2p is responsible for 2-thiolation of the U34 nucleotide, while MSS1p is involved in the C5 modification of 5-carboxymethylaminomethyluridine (cmnm^5^U34) [19], [20]. One aim of this study was to examine whether the nuclear modifier gene *MSS1* modulates the phenotypic manifestation of the yeast C1477G mutant in a YPD medium with neomycin.

The primary limiting factor for growth and reproduction of all biological systems is energy. The energy of cellular bioenergetic systems originates mainly from OXPHOS and glycolysis [21]. Organisms can adjust their metabolism to ensure energy supply and facilitate their growth or survival. A good example is mitochondrial defects can be compensated by an increase of glycolysis in cells for ATP production [22]–[25]. However, the underlying mechanisms of this metabolic shift (mitochondrial to glycolytic) are far from understood. In yeast, the HAP (heme activated protein) complex Hap2/3/4/5p is involved with the respiratory metabolism [26]. The overexpression of Hap4p in aerobic glucose-grown cultures has been reported to positively affect the balance between respiration and glycolysis and towards respiration [27]–[32]. However, at low specific growth rate deletion of *HAP4* had little or no effect compared with the wild-type strain on glucose [33]. Genome-wide genetic analysis has revealed that *HAP5* has a genetic interaction with the glycolytic transcription factor gene *GCR1* [34]. GCR1 has been demonstrated to play a central role in the regulation of glycolysis among the three glycolytic transcription factors RAP1, GCR1 and GCR2 [35]. Whether HAP5 is involved with the mitochondrial to glycolytic shift remains poorly understood.

Here we show that the nuclear modifier gene *mss1* mutation suppresses the neomycin-sensitivity phenotype of the yeast C1477G mutant. We also reveal that glycolysis increasingly compensates for the mitochondrial dysfunction of the *mss1*(P^R^) mutant and insures its growth, in a similar manner to that of the *mto2*(P^R^) mutant [17]. Moreover, we found that the *mss1*(P^R^) strain expressed a significantly high level of *HAP5*. On the basis of the above evidence, yeast strains with disruption or overexpression of *HAP5* were constructed. These strains were characterized by examining growth properties and the gene expression of the glycolytic transcription factors. Thus we identified that in the *mss1*(P^R^) mutant the increase of glycolysis could be mediated by HAP5.

## Materials and Methods

### Yeast strains and culture conditions

The original strains were of the W303-1B strain (*MSS1*(P^S^), α, *ade2*-1, *his3*-1,15, *leu2*-3,*112*, *trp1*-1, *ura3*-1) [12] and the M12-54 strain (*MSS1*(P^R^), a, *ilv5*, *trp2* [ρ^+^, P^R^_454_]) [11]. The *mss1*(P^S^) (α, *ade2*-1, *his3*-1,15, *leu2*-3,*112*, *trp1*-1, *ura3*-1, *mss1::HIS3*) and *mss1*(P^R^) (α, *his3*-1,15, *leu2*-3,112, *ura3*-1, *mss1::HIS3* [P^R^_454_]) strains were constructed by modification as previously described [8], [12], [20]. Standard procedures were used for crossing and selecting diploids, including sporulation and the dissection tetrads [36]. Similarly, the Δ*HAP5*/*MSS1*(P^S^) (α *ade2*-1, *his3*-1,15, *leu2*-3,112, *trp1*-1, *ura3*-1, *hap*5*::HIS3*), Δ*HAP5*/*MSS1*(P^R^) (a *ilv5*, *trp2*, *hap5::ILV5* [ρ^+^, P^R^_454_]), and the Δ*HAP5*/*mss1*(P^S^) (α *ade2*-1, *his3*-1,15, *leu2*-3,112, *trp1*-1, *ura*3-1, *mss1::HIS3*, *hap5::URA3*) strains were constructed from the *MSS1*(P^S^), *MSS1*(P^R^) and *mss1*(P^S^) strains, respectively. The pDB20, an *ADH1* promoter and *URA3* selection marker based yeast multicopy expression vector [37], was used for the expression of *HAP5* in *S. cerevisiae*. The pDB20-ILV5-HAP5 plasmid was constructed by inserting the full-coding region of *ILV5* to *URA3* of pDB20-HAP5 plasmid.

All yeast strains were cultured on YPD complete medium (1% yeast extract, 1% peptone and 2% glucose). The 30 µg/ml neomycin-containing media were prepared by adding 100 mg/ml neomycin stock solutions of antibiotics into YPD after sterilization. 100 mM 2-deoxy-glucose (2-DG) stock solution was prepared in DMSO, and the final concentration of 2-DG in the YPD was 2.5 mM.

### Phenotypic analysis and growth curve

For the phenotypic analysis, yeast strains were inoculated and grown in liquid YPD medium at 30°C overnight until they reached the exponential growth phase. The cultures were harvested and diluted serially to spot on the YPD plates containing neomycin, or neomycin with 2-DG. The plates were incubated at 30°C for 3 days and photographs were taken.

For the growth curve analysis, strains were cultured in liquid YPD both in the absence and presence of 30 µg/ml neomycin and with a starting cell density of 0.01 OD_600_. The growth conditions were monitored by measuring the OD_600_ value every two hours for the first 24 hours.

### Oxygen consumption assay

The oxygen consumption rate (OCR) measurements were carried out as described elsewhere with minor modifications [22]. Yeast cells were cultured overnight at 30°C in a YPD medium until they reached the exponential growth phase. The cell density was adjusted and a 180 µl medium containing cells was seeded in precoated Poly-D Lysine (50 µg/ml) XF 96-well microplates (Seahorse Bioscience) at 4×10^5^ cells per well and then spun down. Following incubations at 30°C for 30 min, the oxygen consumption rate was measured according to the manufacturer's manual on a Seahorse XF96 Extracellular Flux Analyzer in the absence or presence of 30 µg/ml neomycin.

### Northern blotting analysis

Total cellular RNA was obtained from yeast cultures (2.0×10^7^ cells) using a TRIzol Reagent (Invitrogen) according to the manufacturer's instructions. Equal amounts (10 µg) of total RNA were separated by electrophoresis through 1.5% formaldehyde denaturing agarose gel, transferred onto a positively charged membrane (Roche Applied Science) and hybridized with a DIG-labeled *HXK2*-specific antisense RNA probe. The blot was then stripped with stripping buffer (50% formamide, 50 mM Tris/HCl, pH7.5, 5% SDS) and hybridized using *PFK1* and *PYK1* probes, respectively. Finally, the blot was hybridized with a nuclear encoded *ACT1* probe as an internal control [38]. The probes were synthesized from the corresponding plasmid linearized by restriction enzymes using a DIG RNA labeling kit (Roche Applied Science). The plasmids used for preparing the probes were constructed by PCR-amplifying fragments of *HXK2* (positions 24094-24972), *PFK1* (positions 971431-970785), *PYK1* (positions 71983-73069), and *ACT1* (positions 54696-54187), and then cloning the fragments into the pCRII-TOPO vector carrying the SP6 and T7 promoters (Invitrogen).

### Quantitative real-time RT-PCR assay

After the total cellular RNA was isolated, as mentioned above, the RNase-free DNase (Takara Bio Inc., Japan) was added to eliminate DNA contamination according to the manufacturer's instructions. One microgram total RNA was used for the first-strand cDNA synthesis using a PrimeScript RT reagent Kit (Takara). 100 ng cDNA for each sample was used to analyze gene expression with SYBR *Premix EX Taq* (Takara). The cycling program was as follows: an initial cycle of 2 min at 95°C, followed by 40 cycles of 15 s at 95°C, 15 s at 58°C and 20 s at 72°C. The amplification was monitored on an ABI 7900 Real-Time PCR System (Applied Biosystems, USA). *ACT1* was used as an internal control. The PCR primers specific for each gene are listed below (5′-3′): *HXK2*-F, TACGGCTGGACTCAAACCTCA, *HXK2*-R, CGATGATACCAACGGACTTACCT; *PFK1*-F, TCTCTGAAGCAAGCAAGGGTAAG, *PFK1*-R, GAATCTCAAAACCTTAGCGTCAGT; *PYK1*-F, CCCAATCCCACCAAACCAC, *PYK1*-R, TTCTACCAGCGGAGATGACCTT; *RAP1*-F, GTGTAACAACTACCACGCCTTCC, *RAP1*-R, ACTTCACCACCGTTTGCTCTAAT; *GCR1*-F, CAAGTAAGTCGGAGCCCAATG, *GCR1*-R, TAGAAGAACTCTGCGGAAATGATG; *GCR2*-F, TGGATGACGAAGCGGTGG, *GCR2*-R, ACTTTCATTTGGGTGTTATTCGC; *HAP5*-F, TTGGTCAGGGATTGGTGGG, *HAP5*-R, CGGATTCTCGCAAATGGTAAG; and *ACT1*-F, TACTCTTTCTCCACCACTGCTGA, *ACT1*-R, CTTGACCATCTGGAAGTTCGTAG.

### Statistical analysis

All experiments were repeated at least three independent times and the representative data was presented as means ± standard deviation (SD). One-way analysis of variance (ANOVA) was performed to determine the significance between groups. *P<0.05, **P<0.01.

## Results

### Growth properties of yeast strains

**Figure 1:**
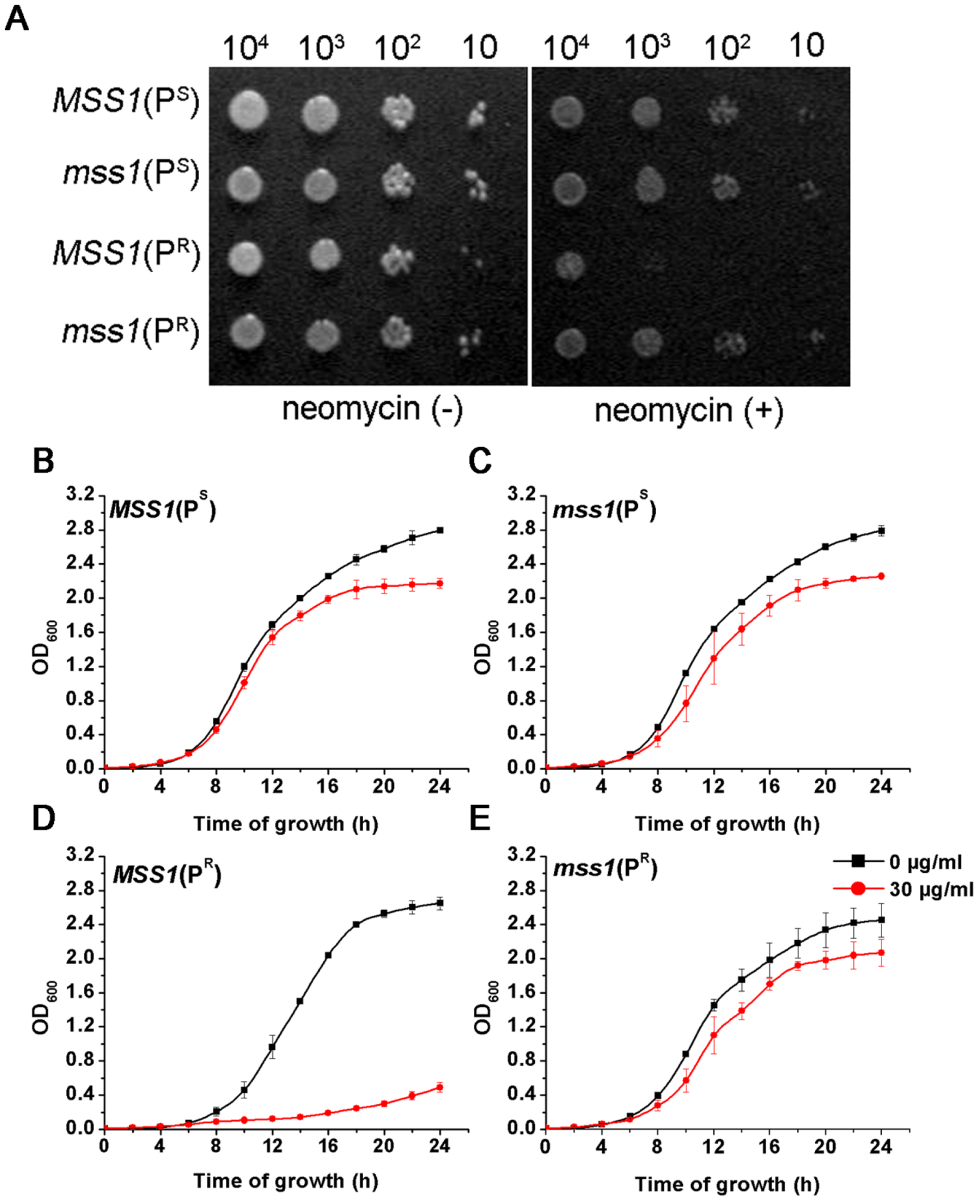
Growth properties of yeast strains. (A) Series dilutions of the four yeast strains *MSS1*(P^S^), *mss1*(P^S^), *MSS1*(P^R^) and *mss1*(P^R^) were spotted on YPD plates in the absence (−) or presence (+) of 300 µg/ml neomycin and incubated at 30°C. The experiment was performed three times with similar results. (B–E) Growth curves of the four yeast strains. Cells from *MSS1*(P^S^), *mss1*(P^S^), *MSS1*(P^R^) and *mss1*(P^R^) strains were cultured in the absence or presence of 30 µg/ml neomycin-supplemented YPD medium and the cell density was determined at different times. Error bars represent standard error from three independent determinations.

The growth properties of yeast strains were determined on a YPD medium in the absence or presence of neomycin (Fig. 1). All of the four strains, *MSS1*(P^S^), *mss1*(P^S^), *MSS1*(P^R^) and *mss1*(P^R^), grew well on YPD agar plates and no significant difference in growth was observed. On the YPD plates with 300 µg/ml neomycin, the growth of *MSS1*(P^R^), the strain carrying the mtDNA C1477G mutation alone, was remarkably suppressed. Interestingly, the growth of the double mutant strain *mss1*(P^R^) changed slightly (Fig. 1A). These phenotypes were further confirmed in a liquid YPD medium without or with 30 µg/ml neomycin (Fig. 1B–E), which is the 90% minimal inhibitory concentration for the *MSS1*(P^R^) strain [17]. When cultured in a YPD liquid medium with neomycin for 16 hours, the growth of the *MSS1*(P^R^) strain was severely inhibited and its OD_600_ value was only about 0.193, whereas the *mss1*(P^R^) strain's OD_600_ value reached about 1.701. Meanwhile, the OD_600_ values for the *MSS1*(P^S^) and *mss1*(P^S^) strains were about 1.987 and 1.910, respectively.

This data indicates that the yeast carrying the mtDNA C1477G mutation (P^R^) was sensitive to neomycin, while the nuclear modifier gene *mss1* deletion suppressed this phenotypic manifestation.

### Mitochondrial respiratory rates

**Figure 2:**
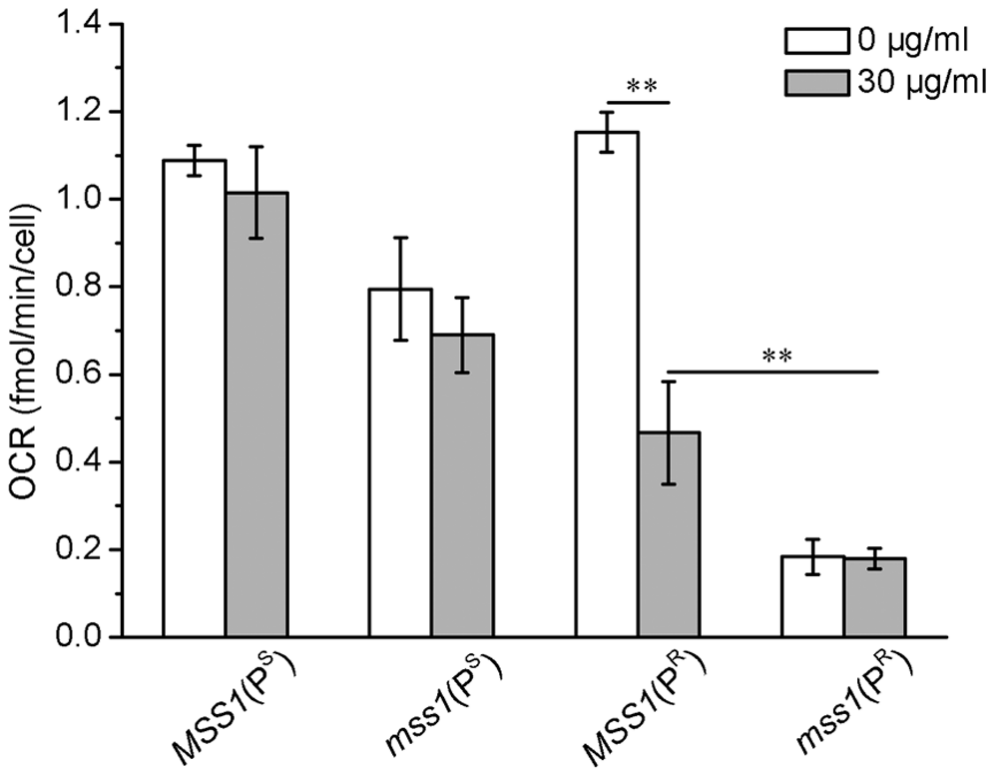
Assay of oxygen consumption rate. The oxygen consumption rate of each yeast strain was measured by a Seahorse FX-96 oxygraph in the absence or presence of 30 µg/ml neomycin. The results are shown as means ± SD of triplicate. **P<0.01.

The oxygen consumption rates (OCRs) of relative strains were measured to determine the yeast mitochondrial function *in vivo* (Fig. 2). The OCR level of the *MSS1*(P^R^) strain decreased significantly (*P*<0.01) from 1.153fMoles/min/cell in YPD medium to 0.467fMoles/min/cell in neomycin-supplemented YPD medium. This represents a reduction of about 59.5%. The OCR of the *mss1*(P^R^) strain was very low both in the YPD medium (0.184fMoles/min/cell) and in neomycin-supplemented YPD medium (0.179 fMoles/min/cell). This data indicates that the interaction between neomycin with the mitochondrial 15S rRNA C1477G mutation (P^R^), or between the *mss1* deletion with the mitochondrial 15S rRNA C1477G mutation can lead to a mitochondrial respiratory defect. The nuclear modifier gene *mss1* deletion may suppress the aminoglycoside-sensitivity of the mtDNA C1477G mutation through inducing other energy compromising pathways, rather than by improving mitochondrial respiration.

### Effect of 2-DG on the growth properties of yeast strains

**Figure 3:**
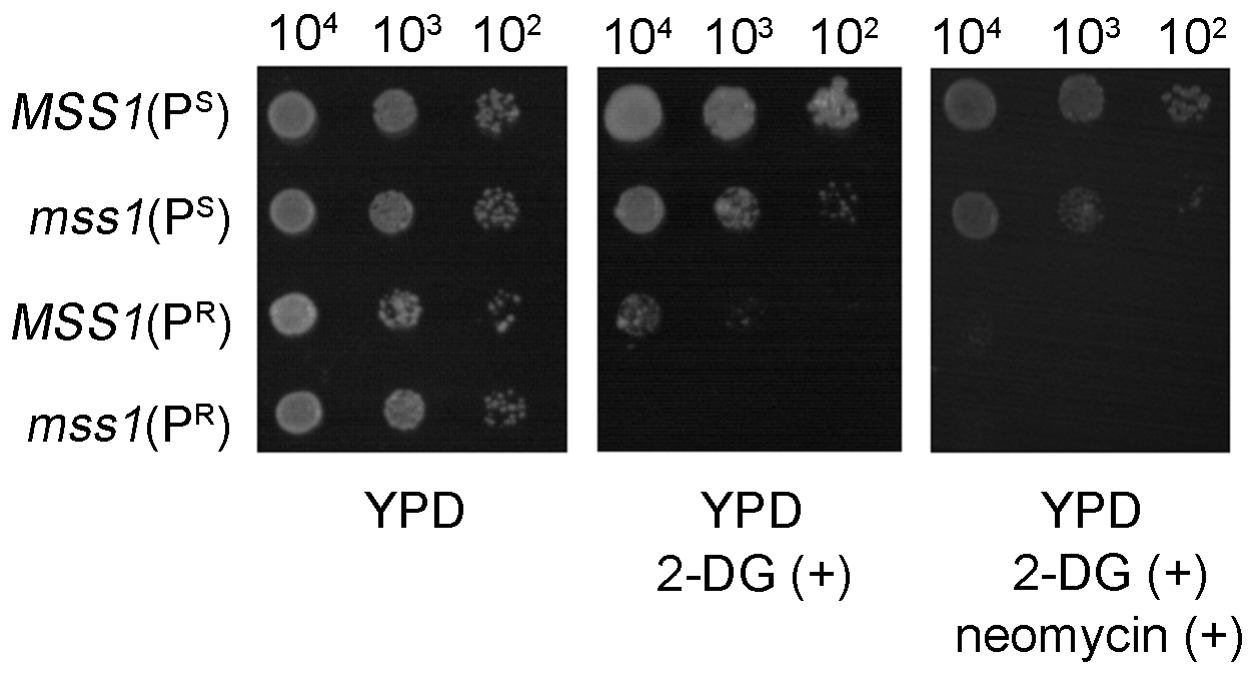
The effect of 2-DG on the growth properties of yeast strains. Series dilutions of the four yeast strains *MSS1*(P^S^), *mss1*(P^S^), *MSS1*(P^R^) and *mss1*(P^R^) were spotted on YPD plates containing 2.5 mM 2-DG, or 2.5 mM 2DG and 300 µg/ml neomycin, and incubated at 30°C. The experiment was performed three times with similar results.

2-DG, as a glucose analogue, competitively inhibits hexokinase and blocks the glycolytic pathway [39]. The double mutant *mss1*(P^R^) strain cannot grow on a YPD medium supplemented with 2.5 mM 2-DG, indicating that it totally depends on the glycolytic metabolism. The *MSS1*(P^S^), *mss1*(P^S^) and *MSS1*(P^R^) strains can maintain their growth through mitochondrial respiration (Fig. 3). This data was consistent with the oxygen consumption rates (Fig. 2). Moreover, it remained the case that no growth was evident for the *mss1*(P^R^) strain on a YPD medium supplemented with 2.5 mM 2-DG and 300 µg/ml neomycin (Fig. 3).

### Transcriptional expression of key glycolytic enzyme genes

**Figure 4:**
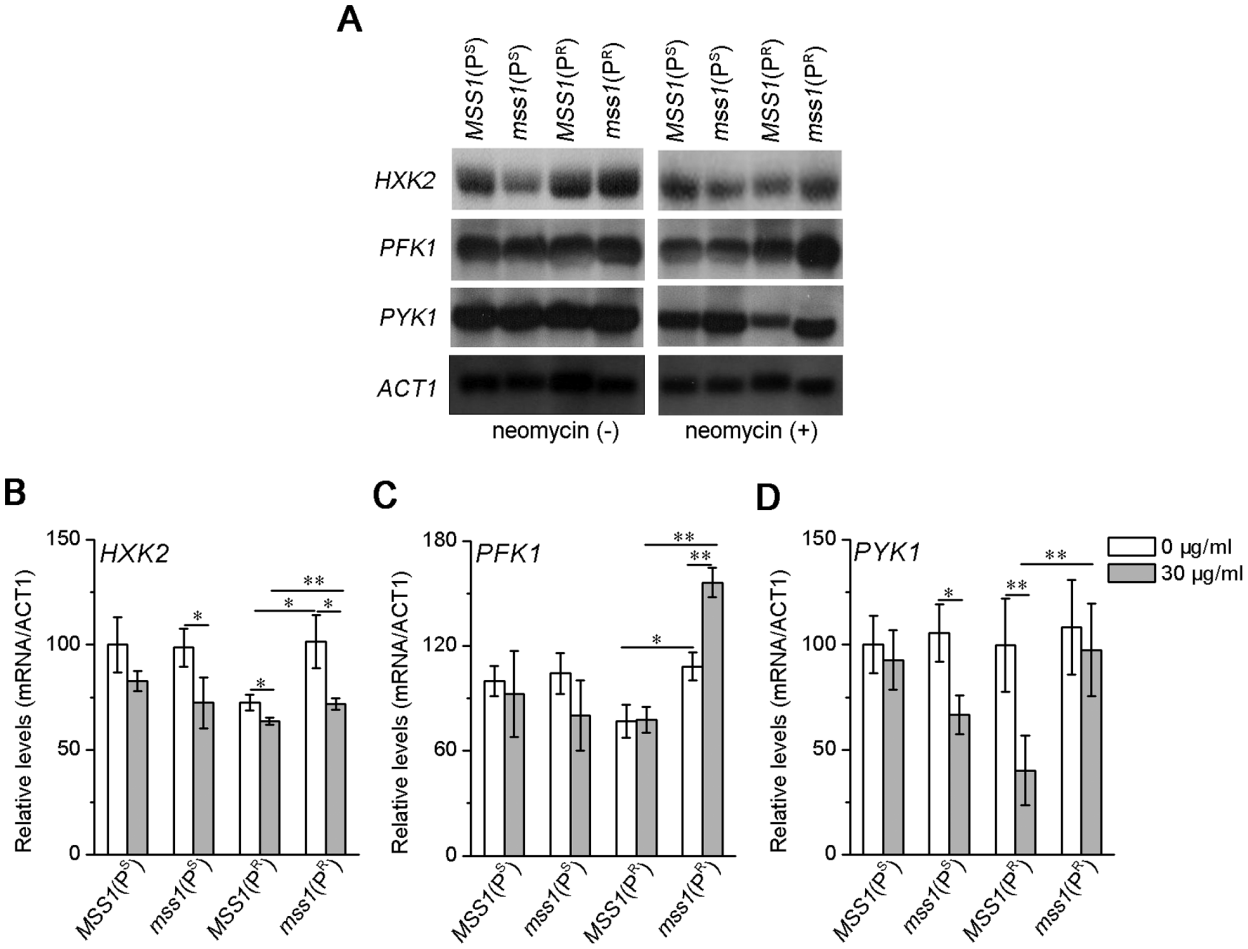
Northern blot analysis of three key glycolytic enzyme genes. (A) Yeast cells were cultured in the absence or presence of 30 µg/ml neomycin for 16 h and harvested. Equal amounts (10 µg) of total cellular RNA from yeast strains were electrophoresed through a 1.5% agarose-formaldehyde gel, transferred onto a positively charged membrane and hybridized first with a DIG-labeled *HXK2*-specific RNA probe. After stripping, the blots were rehybridized with DIG-labeled *PFK1* and *PYK1* probes, respectively. Subsequently, after restripping, they were hybridized with a DIG-labeled *ACT1* probe as an internal control. (B–D) Quantification of the levels of three key glycolytic enzyme genes. The average relative *HXK2*, *PFK1* and *PYK1* RNA content per cell was normalized to the average content per cell of *ACT1*. Values are expressed as percentages of the average values for the wild-type strain *MSS1*(P^S^). Samples from at least three independent cultures of RNA content and *ACT1* for each strain were used in the calculations. *P<0.05, **P<0.01.

Northern blots were used to evaluate the transcript abundance for the three key glycolytic enzymes *HXK2*, *PFK1* and *PYK1*. In a YPD medium, the transcriptional expression of *HXK2* and *PFK1* of the *mss1*(P^R^) strain was significantly higher than that of *MSS1*(P^R^) strain. In the YPD with neomycin medium, the transcript abundance of all three key glycolytic enzyme genes elevated in the *mss1*(P^R^) double mutant. The *PFK1, PYK1* and *HXK2* transcript expression, increased by about 101.3% (*P*<0.01), 143.5% (*P*<0.01) and 12.8% (*P*<0.05) as compared with that in *MSS1*(P^R^) strain, respectively (Fig. 4B–D). To more clearly and quantitatively confirm the changes of *HXK2*, *PFK1*, and *PYK1* expression, we performed the additional experiments by RT-qPCR analysis. In YPD medium, quantitative RT-PCR showed a significant increase of *HXK2* and *PFK1* mRNA in *mss1*(P^R^) strain compared with that of *MSS1*(P^R^) strain. In the YPD with neomycin medium, the transcriptional level of *PFK1*, *PYK1* and *HXK2* in *mss1*(P^R^) strain significantly increased in contrast to that of *MSS1*(P^R^) strain, respectively. This was consistent with Northern blot assay and was shown in Fig. S1 A-C. These data suggests that the nuclear modifier gene *mss1* mutation can lead to an increase of glycolytic capacity for a strain carrying the mtDNA C1477G mutation.

### Transcriptional expression of glycolytic transcription factors

**Figure 5:**
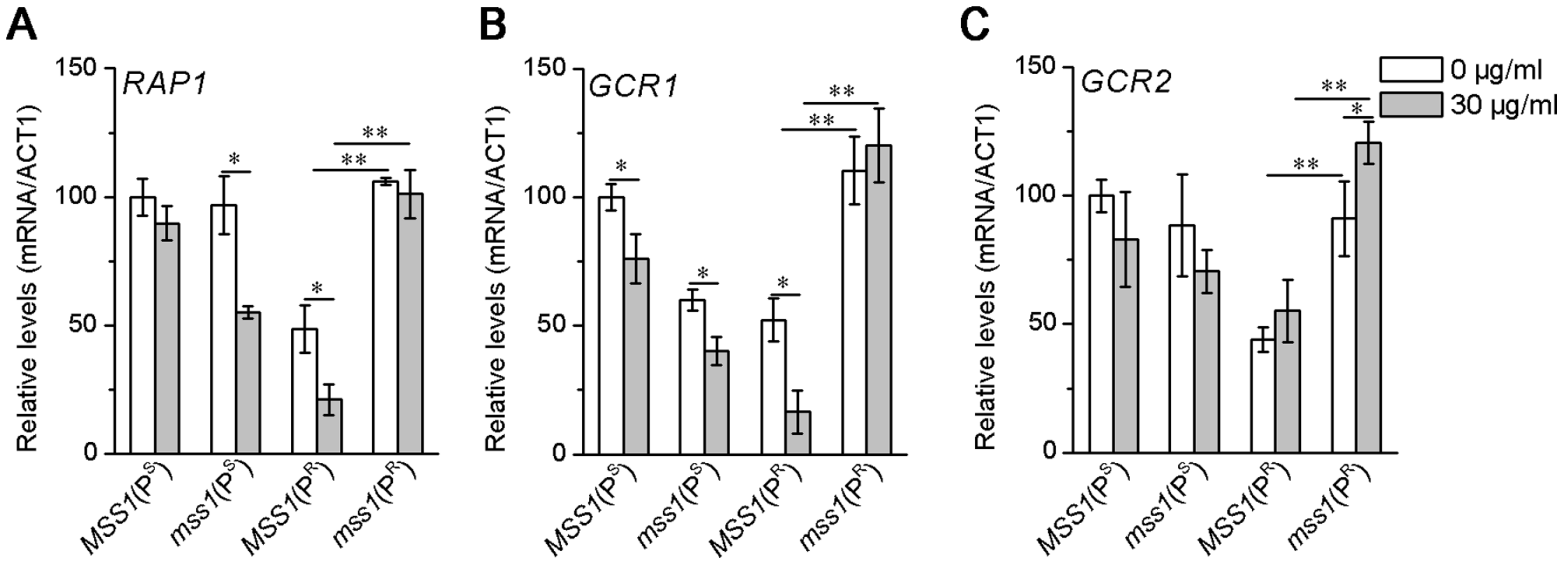
Gene expression of glycolytic transcription factors. Yeast cells were cultured in the absence or presence of 30 µg/ml neomycin for 16 h and harvested. Then RNA was extracted for quantitative RT-PCR analyses. The relative gene expression of *RAP1* (A), *GCR1* (B) and *GCR2* (C) was normalized to the average content per cell of *ACT1*. Values are expressed as percentages of the average values for the wild-type strain *MSS1*(P^S^). Samples from at least three independent cultures of RNA content and *ACT1* for each strain were used in the calculations. *P<0.05, **P<0.01.

The transcripts of glycolytic transcription factors *RAP1*, *GCR1* and *GCR2* were analyzed by quantitative RT-PCR. No matter whether they were in YPD medium or YPD medium with neomycin, the expression levels of *RAP1*, *GCR1* and *GCR2* in the *mss1*(P^R^) strain remained significantly higher than that of the *MSS1*(P^R^) strain. On the other hand, with the exception of *GCR2*, neomycin did not increase the expression levels in the *mss1*(P^R^) strain. Neomycin simply decreased the levels of *RAP1* and *GCR1* in the *MSS1*(P^R^) strain (Fig. 5). This data indicates that the nuclear *mss1* and mitochondrial C1477G mutations act synergistically in increasing the basal levels of *RAP1*, *GCR1* and *GCR2*.

### Gene expression of *HAP5*

**Figure 6:**
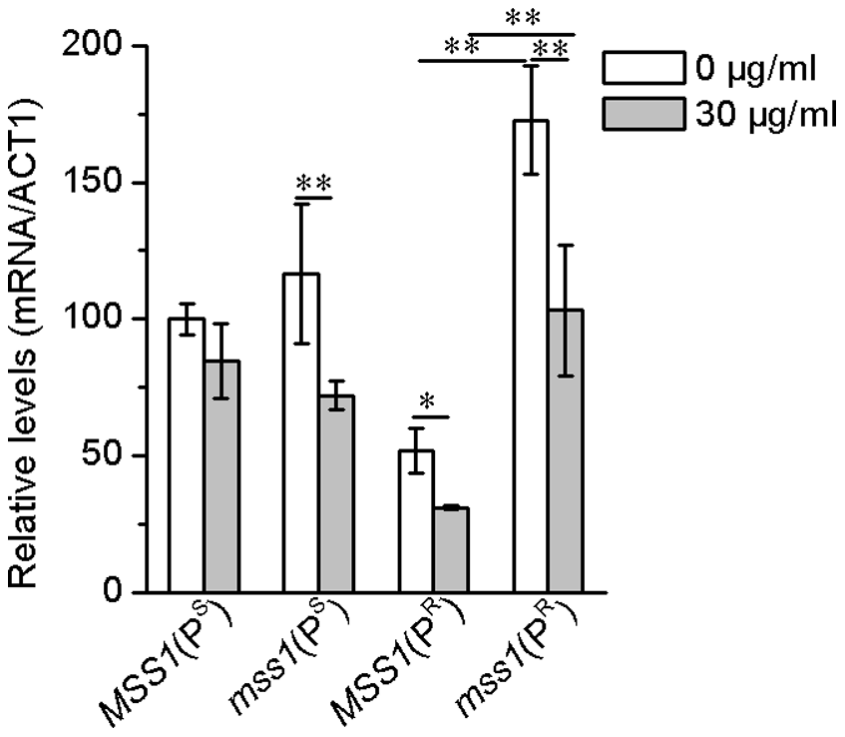
*HAP5* gene expression. Yeast cells were cultured in the absence or presence of 30 µg/ml neomycin for 16 h and harvested. RNA was then extracted for quantitative RT-PCR analyses. The relative gene expression of *HAP5* was normalized to the average content per cell of *ACT1*. Values are expressed as percentages of the average values for the wild-type strain *MSS1*(P^S^). Samples from at least three independent cultures of RNA content and *ACT1* for each strain were used in the calculations. *P<0.05, **P<0.01.

The transcript level of *HAP5* in the yeast strains was determined by quantitative RT-PCR. The *HAP5* transcription level of the *mss1*(P^R^) strain increased significantly compared with that of *MSS1*(P^R^) by about 233.6% (*P*<0.01) in the YPD medium, and 232.9% (*P*<0.01) in the YPD with neomycin medium (Fig. 6). This data indicates that there was a positive correlation between the transcriptional expression of *HAP5* with that of glycolytic genes.

### Effects of *HAP5* on the neomycin sensitivity of *mss1*(P^R^) mutant

**Figure 7:**
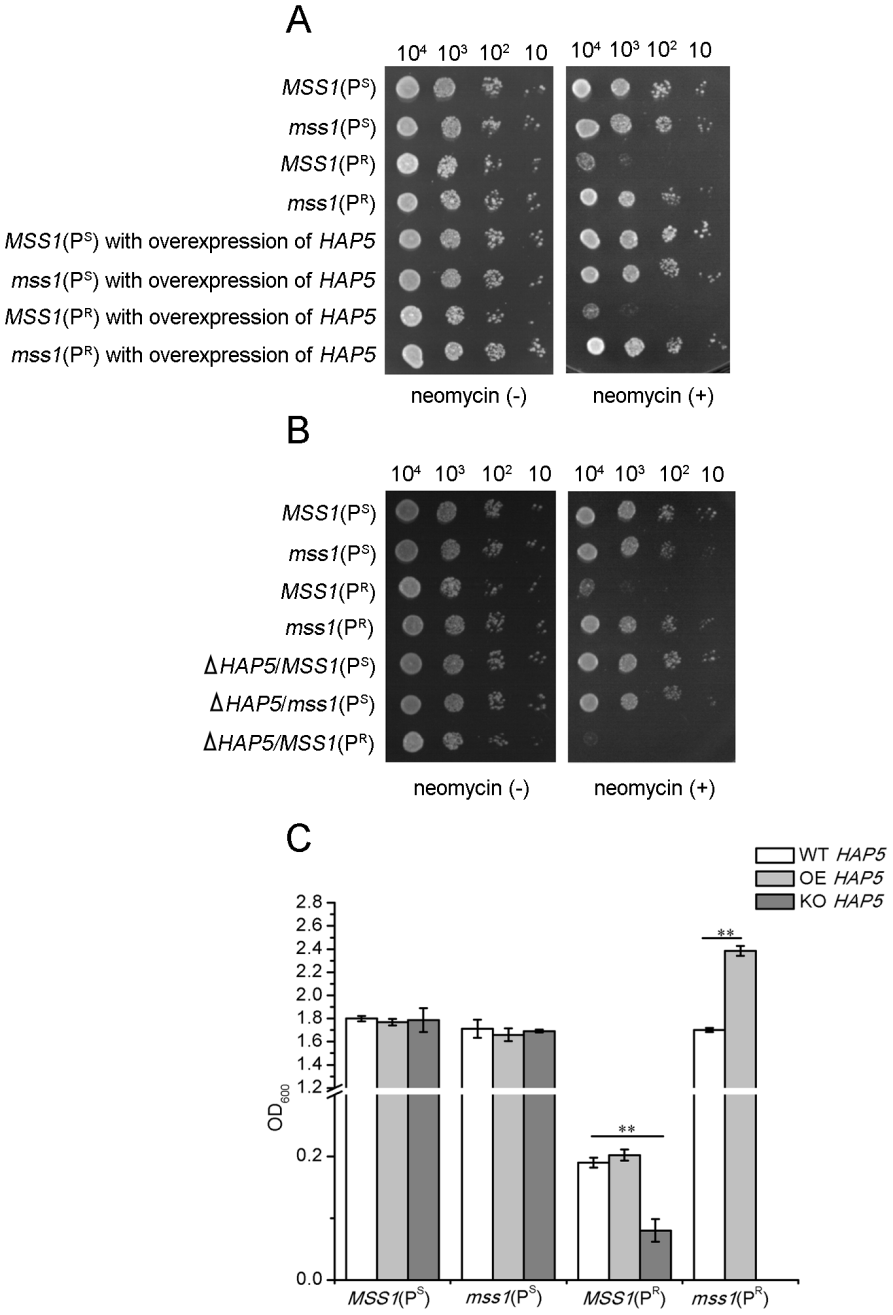
Growth properties of yeast strains with the overexpression or knockout of *HAP5*. (A) Growth properties of wild-type (WT) *HAP5* yeast strains *MSS1*(P^S^), *mss1*(P^S^), *MSS1*(P^R^), *mss1*(P^R^) and their individual *HAP5* overexpression (OE) strains. (B) Growth properties of WT *HAP5* yeast strains *MSS1*(P^S^), *mss1*(P^S^), *MSS1*(P^R^) and their individual *HAP5* knockout (KO) strains. Series dilutions of the yeast strains were spotted on YPD plates in the absence (−) or presence (+) of 300 µg/ml neomycin and incubated at 30°C. The experiment was performed three times with similar results. (C) The yeast cells were cultured in 30 µg/ml neomycin-supplemented YPD medium for 16 h and the cell density was determined. Error bars represent standard error from three independent determinations. **P<0.01.

On neomycin-supplemented YPD plates, the overexpression of *HAP5* in the *mss1*(P^R^) strain resulted in a reduced sensitivity to neomycin compared to the *mss1*(P^R^) strain (Fig. 7A). Moreover, after the cells were cultured in a neomycin-supplemented YPD medium for 16 h, the cell density of the *mss1*(P^R^) strain with the overexpression of *HAP5* increased significantly (*P*<0.01) by about 40%, in contrast to that of the *mss1*(P^R^) strain (OD_600_ value 2.384 *versus* 1.702) (Fig. 7C). These results indicate that *HAP5* has a positive effect on the glycolysis of the *mss1*(P^R^) strain in neomycin-supplemented medium. In contrast to the *MSS1*(P^R^) strain, the knockout of *HAP5* in the *MSS1*(P^R^) strain further enhanced its neomycin sensitivity (Fig. 7B), where the cell density decreased significantly (*P*<0.01) and reduced by about 57.9% in neomycin-supplemented YPD medium after 16 h of culture (OD_600_ value 0.190 *versus* 0.08) (Fig. 7C). Meanwhile, the strain with the *MSS1*, mtDNA C1477G and *HAP5* mutations was not viable from the haploid *mss1*(P^R^) strain. These results suggest that the knockout of *HAP5* has a negative effect on the glycolysis of yeast strains with the mtDNA C1477G mutation. Collectively, the above results indicate that the elevated glycolysis of the *mss1*(P^R^) strain in neomycin-supplemented YPD medium could be mediated by HAP5.

### Gene expression of glycolytic transcription factors in generated *HAP5* yeast strains

**Figure 8:**
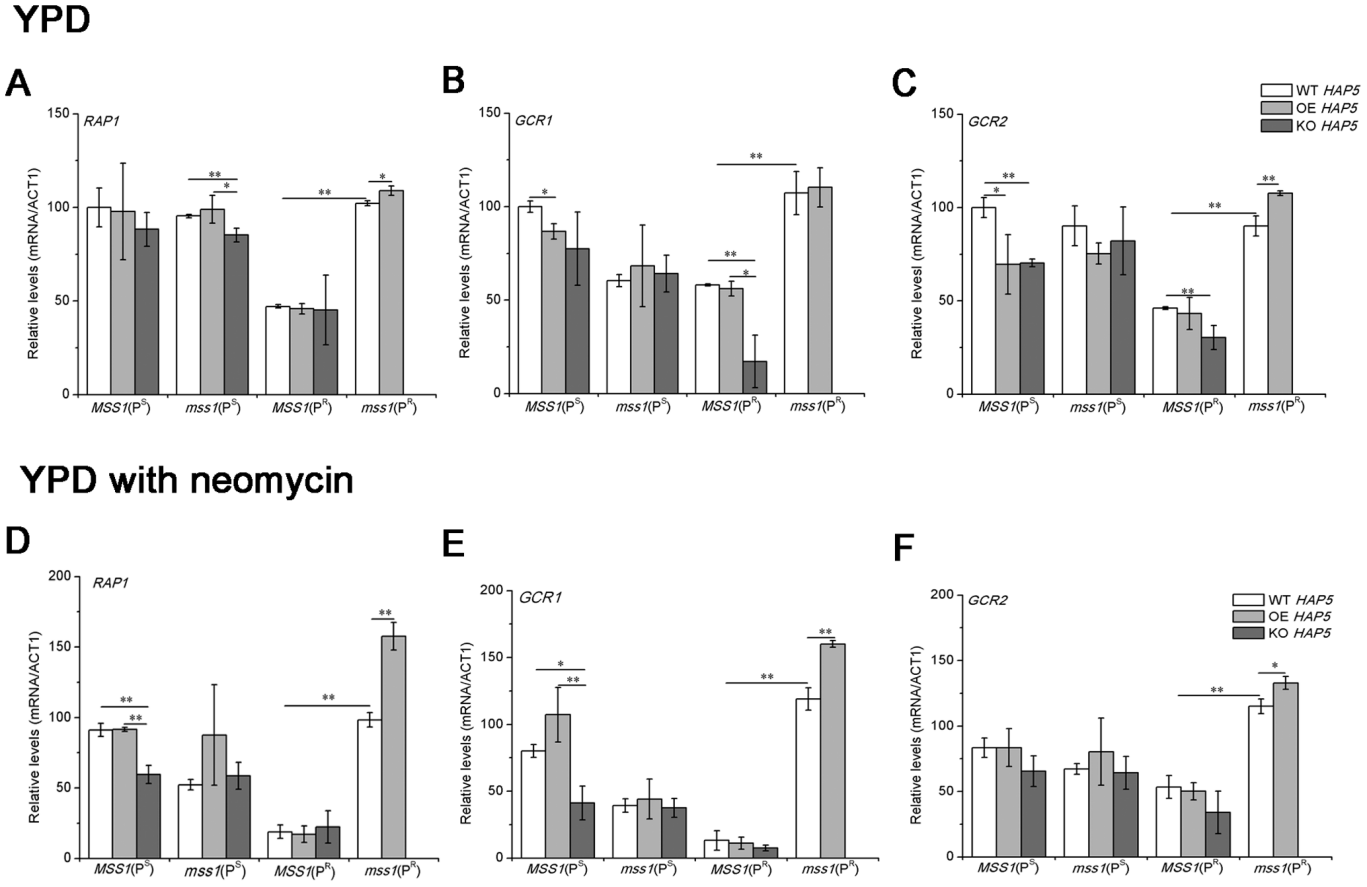
Gene expression of glycolytic transcription factors in overexpression (OE) or knockout (KO) *HAP5* yeast strains. Yeast cells were cultured in a YPD medium (A, B, C) or YPD with 30 µg/ml neomycin (D, E, F) for 16 h and harvested. RNA was then extracted for quantitative RT-PCR analyses. The relative gene expression of *RAP1* (A, D), *GCR1* (B, E) and *GCR2* (C, F) of strains was normalized to the average content per cell of *ACT1*. Values are expressed as percentages of the average values for the wild-type strain *MSS1*(P^S^) in YPD medium. Samples from at least three independent cultures of RNA content and *ACT1* for each strain were used in the calculations. *P<0.05, **P<0.01.

We then detected the expression of glycolytic transcription factors by quantitative RT-PCR in the generated *HAP5* yeast strains. As shown in Fig. 8B and C, in contrast to the *MSS1*(P^R^) strain, the knockout of *HAP5* in the *MSS1*(P^R^) strain led to the expression of the glycolytic transcription factors *GCR1* and *GCR2* being decreased significantly (*P*<0.01) in a YPD medium by about 70.4% and 34.3%, respectively. This suggests that HAP5 is important for the expression and regulation of glycolytic transcription factors in the *MSS1*(P^R^) strain. In addition, the expressions of *RAP1*, *GCR1* and *GCR2* were lower in the *MSS1*(P^R^) and Δ*HAP5*/*MSS1*(P^R^) strains compared to the other yeast strains in a neomycin-supplemented YPD medium (Fig. 8D–F). Compared to that of the *mss1*(P^R^) strain, the overexpression of *HAP5* in the *mss1*(P^R^) strain elevated significantly the expressions of *RAP1*, *GCR1* and *GCR2* by about 60.3%, 34.7% and 11.2% in neomycin-supplemented YPD medium (Fig. 8D–F), respectively, as well as that of *RAP1* and *GCR2* by about 6.7% and 19.5% in YPD medium (Fig. 8A and C), respectively. This data suggests that *HAP5* can up-regulate the expression of the glycolytic transcription factors *RAP1*, *GCR1*, and *GCR2* and stimulate the glycolysis of the *mss1*(P^R^) strain in a neomycin-supplemented medium.

## Discussion

### *mss1* mutation suppressed the neomycin sensitivity of yeast C1477G mutant due to glycolysis increase

Both of our recent works ([17] and present study) clearly showed that in neomycin-supplemented YPD medium, the greatly impaired mitochondrial function of the yeast C1477G mutant was a major cause for its neomycin-sensitivity phenotypic expression (Fig. 2, 3). The mitochondrial function of the *mss1*(P^R^) strain was further impaired when compared with the *MSS1*(P^R^) strain in neomycin-supplemented YPD medium (Fig. 2), suggesting in *mss1*(P^R^) less ATP was produced from the mitochondria. Previous studies have demonstrated that in yeast the transcription factors *RAP1*, *GCR1* and *GCR2* are central to glycolytic gene regulation, and that they act synergistically to allow a high-level of gene expression and promotion of glycolysis [35], [40]–[42]. In the glycolytic pathway, hexokinase (HXK) is involved in the first regulatory step, phosphofructokinase (PFK) in the catalyzing rate-limiting step and pyruvate kinase (PYK) in the final step. These three enzymes catalyze their corresponding irreversible reactions [43], [44]. The up-regulation of *HXK2*, *PFK1* and *PYK1* genes can promote glycolysis in yeast [43], [45]. Our results showed that the expression of those three glycolytic transcription factors had significantly increased in the *mss1*(P^R^) strain, in contrast to *MSS1*(P^R^), no matter whether in YPD or YPD plus neomycin medium (Fig. 5). This was consistent with the expressions of *HXK2*, *PFK1* and *PYK1* (Fig. 4), acting to enhance glycolysis for energy supply. Moreover, the growth of the *mss1*(P^R^) strain could be completely inhibited by 2-DG or a 2-DG and neomycin mixture (Fig. 3). This firmly supported the understanding that the strain depends on the glycolytic pathway to supply energy. Therefore, the nuclear modifier gene *mss1* mutation suppressed the neomycin-sensitivity of the mtDNA C1477G mutant was mainly through a stimulation of glycolysis.

### *HAP5* mediated glycolysis increase of the *mss1*(P^R^) mutant

In this study, the strains with the disruption or overexpression of *HAP5* were constructed to study the shift between respiration and glycolysis. However, we cannot get the Δ*HAP5*/*mss1*(P^R^) deletion strain through six more independent experiments yet, probably this strain was lethal.

The *mss1*(P^R^) strain nearly depended on glycolytic metabolism to ensure its growth. *mss1*(P^R^) expressed the high level of *HAP5* and maintained consistently with the high level of glycolysis (Fig. 2 and 3). The disruption of *HAP5* in *mss1*(P^R^) strain was probably lethal, while the overexpression of *HAP5* in *mss1*(P^R^) increased cell growth and the levels of glycolytic transcription factors (Fig. 7 and Fig. 8), which indicated that HAP5 may play an essential role in the anaerobic respiration. In contrast, the *MSS1*(P^R^) strain mainly depended on mitochondrial respiration for its growth. *MSS1*(P^R^) expressed the low level of *HAP5* and maintained consistently with the low level of glycolysis (Fig. 2 and 3). The disruption of *HAP5* in *MSS1*(P^R^) strain decreased remarkably the expression of *GCR1* and *GCR2*, while the overexpression of *HAP5* in *MSS1*(P^R^) did not increase cell growth and the levels of glycolytic transcription factors (Fig. 7 and Fig. 8), which indicated that HAP5 did not acts as an important factor in aerobic respiration. These data suggested that HAP5 may play a key role to maintain the glycolysis.

Taken together, the comparison between the *MSS1*(P^R^) and the *mss1*(P^R^) strains in neomycin-supplemented medium, showed that the nuclear modifier gene *mss1* mutation led to a significant increase in the steady-state level of *HAP5*, which greatly up-regulated the expression of glycolytic transcription factors and thus stimulated glycolysis. Glycolysis then generated sufficient ATP to compensate for the energy reduction due to the mitochondrial dysfunction. Therefore, the nuclear modifier *mss1* mutation suppressed the neomycin-sensitivity of the yeast C1477G mutant. This study demonstrates that a mtDNA mutation integrates a nuclear gene with environment factors to mediate its phenotypic manifestation. These findings provide a novel insight for us to better understand the phenotypic expression of mtDNA mutations.

## Supporting Information

Figure S1 **Quantitative RT-PCR analysis of key glycolytic enzyme genes.** Yeast cells were cultured in the absence or presence of 30 µg/ml neomycin for 16 h and harvested. RNA was then extracted for quantitative RT-PCR analyses. The relative gene expression of *HXK2* (A), *PFK1* (B) and *PYK1* (C) was normalized to the average content per cell of *ACT1*. Values are expressed as percentages of the average values for the wild-type strain *MSS1*(P^S^). Samples from at least three independent cultures of RNA content and *ACT1* for each strain were used in the calculations. *P<0.05, **P<0.01.
(TIF)
