# Supplementary figures and images for: Mechanistic Study on the Nuclear Modifier Gene MSS1 Mutation Suppressing Neomycin Sensitivity of the Mitochondrial 15S rRNA C1477G Mutation in Saccharomyces cerevisiae
Source: PLoS One. 2014 Mar 3;9(3):e90336. doi: 10.1371/journal.pone.0090336 (PMC3940847; doi:10.1371/journal.pone.0090336)

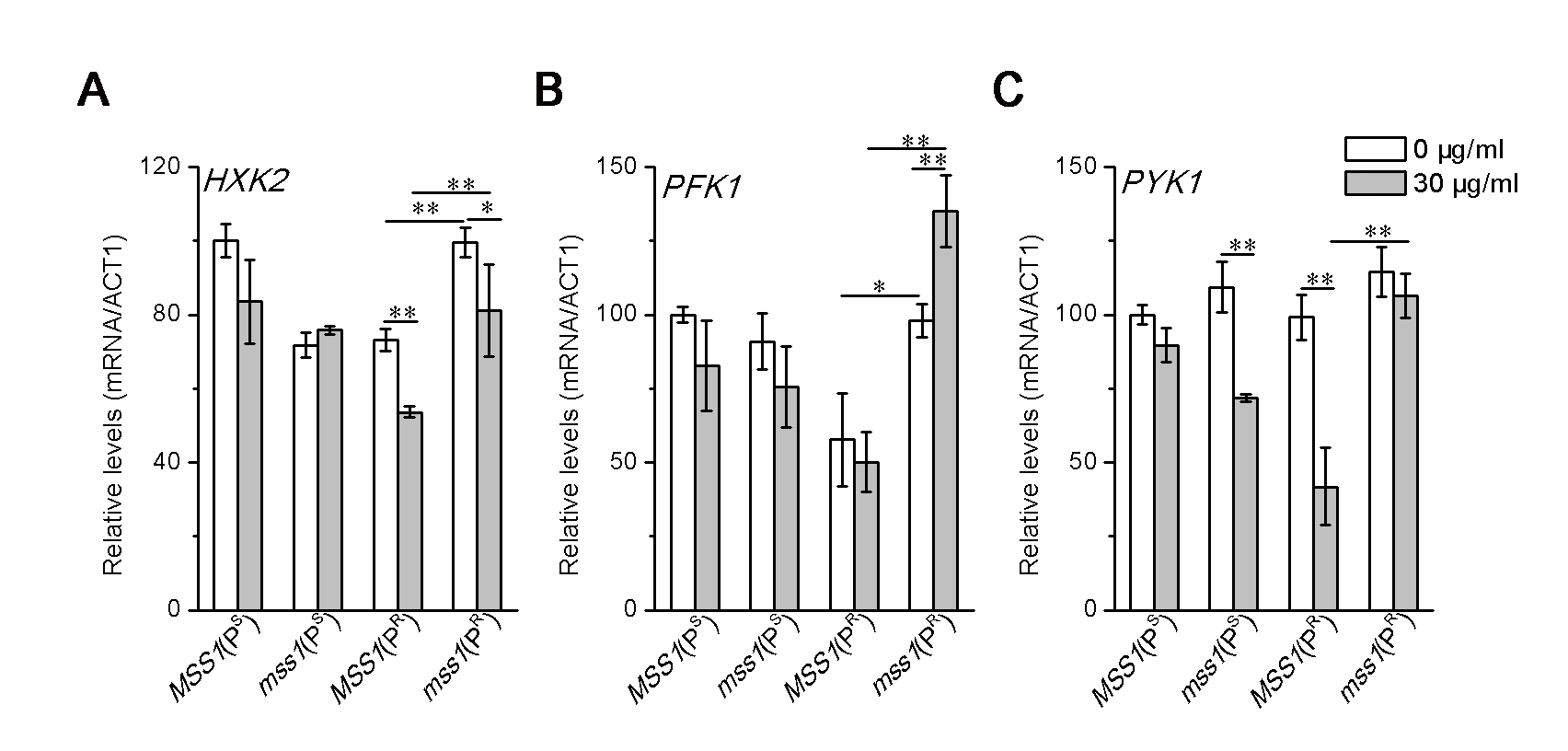

Supplement: Figure S1 — Quantitative RT-PCR analysis of key glycolytic enzyme genes. Yeast cells were cultured in the absence or presence of 30 µg/ml neomycin for 16 h and harvested. RNA was then extracted for quantitative RT-PCR analyses. The relative gene expression of HXK2 (A), PFK1 (B) and PYK1 (C) was normalized to the average content per cell of ACT1. Values are expressed as percentages of the average values for the wild-type strain MSS1(PS). Samples from at least three independent cultures of RNA content and ACT1 for each strain were used in the calculations. *P<0.05, **P<0.01. (TIF) [file pone.0090336.s001.tif]
